# Functions of exosomal non-coding RNAs to the infection with *Mycobacterium tuberculosis*

**DOI:** 10.3389/fimmu.2023.1127214

**Published:** 2023-03-22

**Authors:** Jianjun Wang, Yujie Li, Nan Wang, Jianhong Wu, Xiaojian Ye, Yibiao Jiang, Lijun Tang

**Affiliations:** ^1^ Department of Clinical Laboratory, The First People’s Hospital of Kunshan, Suzhou, China; ^2^ Department of Biochemistry and Molecular Biology, School of Life Sciences, Central South University, Changsha, China

**Keywords:** exosomes, non-coding RNAs, *Mycobacterium tuberculosis*, tuberculosis, biomarkers

## Abstract

Tuberculosis (TB) is a major infectious disease induced by *Mycobacterium tuberculosis* (*M. tb*) which causes the world’s dominant fatal bacterial contagious disease. Increasing studies have indicated that exosomes may be a novel option for the diagnosis and treatment of TB. Exosomes are nanovesicles (30-150 nm) containing lipids, proteins and non-coding RNAs (ncRNAs) released from various cells, and can transfer their cargos and communicate between cells. Furthermore, exosomal ncRNAs exhibit diagnosis potential in bacterial infections, including TB. Additionally, differential exosomal ncRNAs regulate the physiological and pathological functions of *M. tb*-infected cells and act as diagnostic markers for TB. This current review explored the potential biological roles and the diagnostic application prospects of exosomal ncRNAs, and included recent information on their pathogenic and therapeutic functions in TB.

## 1 Introduction

Tuberculosis (TB) is a severe infectious disease that is still the major cause of death from infection, regardless of worldwide progress in health and diseases (1, 2). Recent estimates from a new World Health Organization report show that ~1/4 of the international population are infected with *Mycobacterium tuberculosis* (*M. tb*), which leads to 1.4 million deaths per year (3, 4). Pathogenically, *M. tb* can survive for a long time in macrophages of tubercle granulomas in the human host (5). Macrophages are key components of the host innate immune responses to *M. tb* that could eliminate mycobacteria *via* different mechanisms, including apoptosis, immune-inflammatory responses and phagocytic activity (6).

The diagnosis and analysis of TB are mainly based on TB culture or PCR. Usually, sputum smear microscopy is the most extensively used; however, regardless of its high specificity, its low sensitivity restricts its diagnostic value (7). However, TB cultures take nearly 42 days to detect identifiable growth, thus the long culture period limits its clinical diagnosis (8). At present, the Xpert MTB/RIF and ultra-version tests detect genetic material from *M. tb* as sensitively as microbial cultures (9). These assays are rapid but expensive and widely unavailable (10, 11), and call for better diagnostic technologies for *M. tb*. Therefore, sensitive and specific diagnostic assays are important in controlling and preventing infections from spreading, and novel biomarkers are urgently required due to these problems of current TB diagnostics (12, 13).

In recent years, exosomes, small vesicles derived from various cells, have shown great potential as diagnostic markers and in treatment depending on the cargo inside. Furthermore, it has been indicated that exosomal non-coding RNAs (ncRNAs) are critical regulators involved in the immune defense of *M. tb* infection. Exosomal ncRNAs regulate the host to resist *M. tb* infection by dominating the relevant signaling pathways in the infection process (14). With the development of sequencing technology, a large number of exosomal ncRNAs have been identified in different biological processes of TB. More and more studies have demonstrated that exosomal ncRNAs regulate host gene expression at the level of transcription and post-transcription, which is closely related to the adaptation of TB to the host environment and the generation of pathogenicity (15). These exosomal ncRNAs interact with each other, as well as other components, including proteins and DNA, thus affecting the occurrence and development of TB.

The present study comprehensively reviews the function and molecular mechanisms of exosomal ncRNAs in the physiological and pathological process of *M. tb* infection.

## 2 The biological functions of exosomes

**Figure 1:**
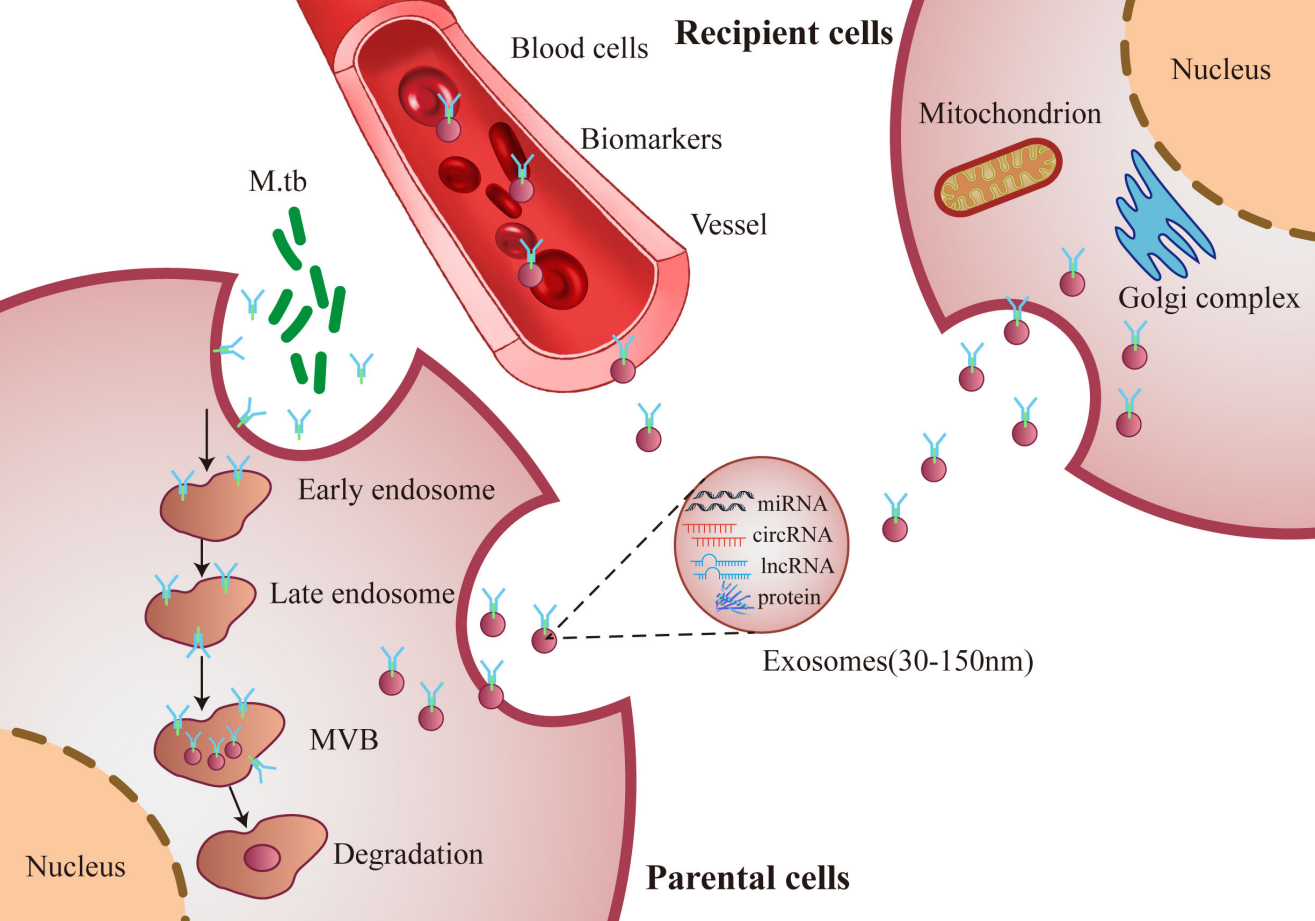
Schematic diagram of secretion synthesis and transmission of exosomes. M. tb infected host cells forms and secretes exosomes, which transmit biological information and regulate the functions between cells through exosomes components, such as miRNA, circRNA, lncRNA and proteins.

Exosomes are 30-150 nm microvesicles, have the same topology as their origin cells and exhibit enrichment of selected proteins, lipids and nucleic acids (16, 17). Exosomes can be distinguished from other extracellular structures (ectosomes and apoptotic blebs) due to their size and the protein factors intercalated in their membranes (18). Exosomes originate from almost all cell types, are separated from nearly all human biofluids and carry functional molecules, including nucleic acids (mRNA, ncRNAs and DNA), proteins, metabolites and lipid modulators (19–22). Exosomes are pivotal in cell-cell communication and transfer biological information by shuttling their cargo to either local or distant cells, and thereby, modulate the function of the recipient cells (**Figure 1**) (23–25). Exosomes are regarded as novel diagnostic biomarkers under various pathological environments, including cancers and infections (26–29). Exosomal contents are employed as signatures of various cancers and infectious diseases (30).

Although exosomes involved in cell-to-cell communication and immune regulation are a topic of intense research, most reports divert to finding aberrations in exosomal protein and RNA contents related to disease occurrence and advancement, which are initially associated with cancer or infection (31). In particular, microRNAs (miRNAs/miRs) are transferred by exosomes that participate in the initiation of various respiratory system diseases, including TB, in which they are critical in inflammation and pathogenesis (32, 33). The miRNA profiles expressed differently in different pathological environments suggest that microvesicles may be related to the occurrence of diseases (34). It is well-known that exosomes induce a series of immune responses in *M. tb* infection.

It has been reported that *M. tb* could induce macrophages to secrete exosomes with pathogen-associated molecular patterns, and these exosomes are in turn transferred into uninfected macrophages to be activated through the Toll-like receptor and myeloid differentiation factor 88-dependent pathway (35, 36). Notably, exosomes from T cells are transferred into dendritic cells (DCs) and induce more resistant DC antiviral responses *via* the cyclic GMP-AMP synthase/stimulator of interferon response cGAMP interactor-1 cytosolic DNA-sensing pathway and *via* the expression of interferon regulatory factor-3-dependent interferon-regulated genes (37). Notably, exosomes may be a candidate vector for vaccines or treatment. Exosomes carrying mycobacterial antigens can protect mice against *M. tb* infection, indicating the potential of exosomes in serving as a novel cell-free vaccine targeting *M. tb* infection (38). Exosomes from *M. tb*-infected bone marrow-derived macrophages could induce autophagy for *in vitro M. tb* killing and also decreased the mycobacterial burden in the lungs of mice with low tissue damage (39). These findings indicate that exosomes not only induce the immune response but can also be used as a vaccine. However, the detailed immunological function of exosomes during *M. tb* infection is still to be revealed.

The present review describes the discovered roles of exosomes in TB disease, the translation work in vaccine development and how to govern the circling of these dynamic vesicles for diagnostic aims. In addition, exosomal RNAs (especially miRNAs) may be a perfect platform to generate diagnostic biomarkers because the exosomes-regulated resistance against RNA degradation is durable (40).

## 3 The functions of exosomal ncRNAs

NcRNAs are non-protein coding molecules with <200 nucleotides, and comprise a myriad of RNA classes, including miRNAs, circular RNAs (circRNAs; circs) and long non-coding RNAs (lncRNAs), with diverse functions, including numerous small transcripts without related functions (41, 42). Exosomes derived from cells in different physiological states contain various ncRNAs, and the composition and species of ncRNAs differ markedly.

Expression assays demonstrate that ncRNAs are accurately modulated in space and time, and a disturbance in gene expression modes can lead to pathological phenotypes, including various cancers and infectious diseases (43). Among ncRNAs, miRNAs are the most-studied and participate in post-transcriptional modulation. MiRNAs are largely conserved ncRNAs that could modulate gene expression by base-pairing. Therefore, variations in transcriptional output from these molecules reflect anomalies in global transcription in cells. Additionally, miRNAs can be delivered into or out of cells by vesicles, which could be detected in body fluids (44). In addition, miRNA profiles can be related to disease development or division among diseases with identical symptoms (45, 46), demonstrating their clinical significance.

Accumulating studies have demonstrated that exosomal ncRNAs are associated with various diseases, including diabetes, coronary artery disease, cancers and infectious diseases (47–49). For instance, Lai et al. reported that exosomal lncRNA SOX2 overlapping transcript (SOX2-OT) promoted ovarian cancer progression, and SOX2-OT, miR-181b-5p and stearoyl-CoA desaturase 1 may serve as potential targets for the treatment of ovarian cancer (50). However, in breast cancer, Zhao et al. found that exosomal transfer of miR-181b-5p conferred senescence-mediated doxorubicin resistance by modulating BCL2 associated transcription factor 1 (51). These studies indicate that exosomal ncRNAs are altered in diseases, and the regulatory targets involved by exosomal ncRNAs are also different.

Exosomes transport ncRNAs to communicate phenotypic characteristics between cells (52). Some potential biomarkers (human miRNAs, mRNAs and mycobacterial RNAs in exosomes) can be used for the identification of TB (53). A new issue in molecular biology is that fluids (e.g., blood or urine) are used to substitute invasive biopsy in disease prognostics and diagnosis. These body fluids carry large protein-covering lipoprotein composites (e.g., exosomes), and DNA and RNA molecules, and are actively derived by living cells (54). The genetic substances covered in exosomes are stable and undegradable and can be used to noninvasively detect both chronic and infectious disorders (55, 56). However, there is no profound exosomal RNA-sequencing (RNA-seq) in human clinical samples of patients with active TB (ATB) or latent TB infection (LTBI).

## 4 The function of exosomal miRNAs in *M. tb* infection

Exosomes mediate the exchange of intricate intercellular messages such as miRNAs (57). miRNAs are small 18-22-nt RNAs that are pivotal in mediating gene expression and translation. miRNAs impact most biological functions, and their dysregulation is related to several pathologies (58). Functional miRNAs may be embedded in exosomes and delivered to target cells, regulating the roles of recipient cells by affecting their transcriptome and/or proteome (59). Additionally, exosomal miRNAs can exist stably in body fluids, and are associated with information of maternal tissues or cells depending on miRNA expression and composition (60, 61).

MiRNAs are involved in the mediation of inflammatory procedures amid *M. tb* infection (62, 63). *M. tb* infection triggers various physiological responses in infected cells, resulting in host immune abnormality and metabolic re-patterning (64). This regulation of host cell reactions allows bacteria to isolate vital host factors to meet their material and energy demand to facilitate intracellular survival (65). These steps may be managed by subversing host miRNA networks involved in the mediation of carbon, nitrogen and lipid metabolism in the infected cells (66, 67).

Exosomes are critical extracellular antigen sources and can promote T cell immunity after *M. tb* infection in mice (68). Generally, exosomes are promising for diagnosis, treatment or vaccine invention, and exosome-based treatment for patients with TB should be further studied (69). Recent research shows that exosomal miRNAs are potential diagnostic tools for TB. The present review summarizes and elaborates the information on the pathogenic and therapeutic functions and roles as diagnostic biomarkers of exosomal miRNAs in TB (**Table 1**).

**Table 1:** Summary of exosomal miRNAs from *M. tb* infected subjects (*in vivo* or *in vitro*).

Number	miRNA	Samples	Method screening	Expression pattern	Refs
1	miR-100-5p, miR-3473b, miR-24-3p, miR-210-3p, miR-423-5p, miR-5100, miR-27-3p, miR-223-5p, miR-99a-5p, let-7d	Macrophages	qRT-PCR	decrease	(53)
2	miR-223, miR-424, miR-302a, miR-21, miR-520d-3p, miR-486-5p, miR-451, miR-550, miR-421, miR-640, miR-144, miR-329	PBMC	miRNA microarray	increase	(70)
3	miR-130b, miR-342-5p, miR-155, miR-181b, miR-548b-3p	PBMC	miRNA microarray	decrease	(70)
4	miR-205-5p, miR-200c-3p, miR-429, miR-200b-3p, miR-200a-3p, miR-203a-3p, miR-141-3p, miR-148a-3p, miR-451a, miR-150-5p	TPE	qRT-PCR	increase	(71)
5	miR-483-5p, miR-375	TPE	qRT-PCR	decrease	(71)
6	miR-186-5p, miR-142-3p, miR-493-3p, miR-17-5p, miR-335-3p, let-7e-5p, miR-185-5p, miR-146b-5p, miR-486-5p, miR-192-5p, miR-223-3p, miR-222-3p	THP-1 macrophages	RNA sequencing	increase	(72)
7	miR-548o-3p, miR-126-5p	THP-1 macrophages	RNA sequencing	decrease	(72)
8	miR-27b-3p, miR-93-5, miR-25-3p, miR-1198-5p, let-7c-5p, let-7a-5p, miR-7658-5p, miR-7069-5p, miR-8092, miR-98-5p, miR-212-3p, miR-181b-5p, miR-3057-5p, miR-203-3p, miR-6516-5p, miR-181d-5p, miR-30a-3p, miR-1933-3p, miR-148b-5p, miR-99b-3p	RAW264.7	RNA sequencing	increase	(73)
9	mir-7018-p5, miR-194-5p, miR-301b-3p, miR-5110, miR-144-3p, miR-874-3p, miR-363-3p	RAW264.7	RNA sequencing	decrease	(73)
10	miR-484, miR-425, and miR-96	Serum	qRT-PCR	increase	(74)
11	miR-191, miR-20b, miR-26a, miR-106a,let-7c, miR-20a, miR-486	Plasma	RNA sequencing	increase	(75)
12	miR-3128, miR-1468, miR-3201, miR-8084	Plasma	RNA sequencing	decrease	(75)
13	miR-96, miR-96, miR-1293, miR-4467, miR-6848, miR-6849, miR-4488, miR-425, miR-4732, miR-484, miR-5094	PBMCs	RNA sequencing	increase	(76)
14	let-7, miR-155, miR-146a, miR-145, miR-21	PBMCs	RNA sequencing	unknown	(77)
15	miR-125a-5p, miR-143-3p, miR-210-3p, miR-23b, miR-17, miR-181b-5p, miR-320a,	Serum	RNA sequencing	decrease	(78)
16	miR-20a-5p, miR-584	Serum	RNA sequencing	increase	(78)
17	let-7e-5p, let-7d-5p, miR-450a-5p, miR-1246, miR-2110, miR-370-3p, miR-629-5p, miR-140-3p, miR-146-5p, miR-103a-3p, etc.	Serum	RNA sequencing	increase	(79)
18	miR-381-3p, miR-133a-3p, miR-127-3p, miR-381-3p, miR218-5p, miR-206, etc.	Serum	RNA sequencing	decrease	(79)
19	miR-197-3p, let-7e-5p, miR-223-3p	Serum	RNA sequencing	increase	(80)

It has been reported that 57 exosomal miRNAs were found in *M. tb*-infected macrophages (e.g., Mmu-223 and 486-5p) and most of these miRNAs were decreased (53). The suppression of these exosomal miRNAs was evaluated by the quantified miRNAs for mRNA targets using miRDB and functional Kyoto Encyclopedia of Genes and Genomes (KEGG) pathway analysis, and the results indicated that potential gene targets for these miRNAs included those associated with immune surveillance and inflammation. These results imply the richness of cell miRNAs after *M. tb* infection and reveal immune mechanisms induced by the pathogen. Wang et al. detected 17 exosomal miRNAs differentially expressed (DE) in peripheral blood mononuclear cells (PBMCs) in patients with ATB, patients with LTBI and healthy controls, respectively, and these exosomal miRNAs were involved in functions in hematopoietic cell differentiation and the transition from LTB to ATB (70). Among these 17 differential miRNAs, six miRNAs (miR-21, miR-223, miR-302a, miR-424, miR-451 and miR-486-5p) were upregulated and miR-130b exhibited reduced expression in patients with ATB; and four miRNAs (miR-144, miR-365, miR-133a and miR-424) were upregulated and three miRNAs (miR-500, miR-661 and miR-892b) were downregulated in patients with ATB (70). However, miR-424 was upregulated in patients with ATB compared with patients with LTBI and healthy controls (70). Therefore, mycobacterial infection of macrophages led to general inhibition of miRNA incorporation into exosomes and exosomal miRNA released from *M. tb*-infected macrophages may have a potential function in diagnosis during mycobacterial infection.

Wang et al. reported that miR-148a-3p, -451a and -150-5p were all upregulated with different fold changes in pleural effusions (PEs) of tuberculosis and other benign lesions using RNA-seq and reverse transcription-quantitative PCR (RT-qPCR). These different miRNA profiles may support the use as biomarkers for differential diagnosis of PEs with more verification based on larger cohorts (71). As reported, 495 exosomal miRNAs related to TB infection were found using whole transcriptome high-throughput sequencing, and it was identified that miR-185-5p, miR-146b-5p and miR-17-5p were increased in exosomes (72). Interestingly, miR-185-5p was markedly increased in all the following samples, including bacille Calmette-Guerin (BCG)-infected monocytes from PBMCs and exosomes from patients with TB (72). These differential miRNAs are excellent biomarkers for the infection of TB. Zhan et al. found 20 upregulated and seven downregulated exosomal miRNAs in *M. tb*-infected macrophages, of which miR-27b-3p, miR-93-5p, miR-25-3p, miR-1198-5p, let-7c-5p and let-7a-5p were considerably upregulated based on high-throughput sequencing (73). A bioinformatics experiment implied that these DE exosomal miRNAs were engaged in several bioprocesses and pathways, and the target genes of the top six miRNAs in the upregulated group were positively related to apoptosis modulation (73). The miRNA expression profile in macrophage exosomes differed after BCG infection, and the DE miRNAs participated in multiple bioprocesses and pathways (73).

Alipoor et al. identified miR-484, miR-425 and miR-96 in serum-derived exosomes of patients with TB by RT-qPCR, and these three miRNAs were markedly increased in the serum, suggesting that these exosomal miRNAs have diagnostic potential in ATB, and their diagnostic value could be improved through combination with conventional diagnostic markers (74). Hu et al. identified six exosomal miRNAs (miR-20a, miR-20b, miR-26a, miR-106a, miR-191 and miR-486) that were increased, and four miRNAs (miR-3128, miR-1468, miR-3201 and miR-8084) that were decreased in patients with TB using RT-qPCR (75). These exosomal miRNAs combined with health records could facilitate clinical discovery of TB meningitis and pulmonary TB (PTB) (75). Alipoor et al. detected 11 exosomal miRNAs (miR-1224, miR-1293, miR-425, miR-4467, miR-4732, miR-484, miR-5094, miR-6848, miR-6849, miR-96 and miR-4488) that were upregulated in BCG-infected monocyte-derived macrophages (MDMs), and these miRNAs were engaged in some key pathways, such as central C metabolism, fatty acid and sugar metabolism, amino acid metabolism, bacterial invasion pathways, and cell pathways (76). These exosomal miRNAs reflect the host-pathogen interaction and subversion of host metabolic processes following infection (76). Mortaz et al. reported that the infection of MDMs with BCG led to the release of a number of exosomal miRNAs, including let-7 family members, and miR-155, miR-146a, miR-145 and miR-21, all of which could target critical immune genes and pathways in BCG-infected MDMs (77). However, these results need to be verified and the presence of these miRNAs in the blood should be tested further to estimate their specificity and selectivity as a diagnostic tool in patients with TB.

Guio et al. found three downregulated miRNAs (miR-143-3p, miR-210-3p and miR-20a-5p) and one upregulated miRNA (miR-20a-5p) for LTB, and three decreased miRNAs (miR-23b, miR-17 and miR-181b-5p) and one increased miRNA (miR-584) for ATB using small RNA-seq, and only two miRNAs were shared by the two types of TB (miR-125a-5p and miR-203a) (78). These exclusive miRNAs are promising regulators of common or exclusive KEGG pathways related to infectious disorders, cancers and immunology. Lyu et al. identified 250 exosomal miRNAs, including 85 specifically expressed miRNAs in serum exosomes of patients with LTBI or TB and healthy controls using small RNA-seq (79). Among the 250 DE miRNAs, 49 upregulated (e.g., miR-146-5p, miR-103a-3p and miR-103b) and 21 downregulated (e.g., miR-381-3p, miR-133a-3p and miR-127-3p) miRNAs were detected for LTBI, and 37 upregulated (e.g., miR-629-5p, miR-140-3p and miR-151a-3p) and 10 downregulated (e.g., miR-381-3p, miR-128-3p and miR-218-5p) miRNAs were identified for TB (79). In addition, they identified 18 and 67 specifically expressed miRNAs in the LTBI (e.g., let-7e-5p, let-7d-5p, miR-450A-5p) and TB (e.g., miR-1246, miR-2110 and miR-370-3p) groups. These findings provide a critical reference and better understanding about miRNAs and repetitive region-obtained small RNAs in exosomes amid *M. tb* infection, and promote the generation of potential molecular targets for the diagnosis of LTB or ATB (79). In addition, Lyu et al. used RNA-seq for a limited RNA library to test divergent exosomal miRNA modes in sera of healthy individuals, and patients with LTBI and TB, and revealed six exosomal miRNAs, and three continually increased miRNAs, including miR-3184-5p, miR-140-3p and miR-423-3p, were detected as potential regulators in TB advancement (79). Additionally, they both assessed the DE miRNA mode with DE mRNA modes. They reported relevant data on the potential roles of exosomes in the whole *M. tb* contagion and suggested that the identified exosomal miRNAs may be used as biomarkers for diagnosis. Interestingly, various literatures show that the types and abundance of exosomal ncRNAs in active and latent tuberculosis are very different, and there are some specific ncRNAs related to tuberculosis progress. Therefore, exosomal ncRNAs can be used to distinguish active and latent tuberculosis.

Carranza et al. found three upregulated miRNAs (miR-let-7e-5p, miR-197-3p and -223-3p) to be sensitive separators between controls and patients with TB for both drug-resistant-TB and multidrug-resistant (MDR)-TB groups using a multivariate analysis (80). MiR-let-7e-5p was upregulated in the MDR-TB group without type 2 diabetes mellitus (T2DM), indicating that miR-let7e-5p is a possible biomarker for the detection and treatment of MDR-TB without T2DM (64). MiRNAs in exosomes, which are exhaled, have been suggested as potential biomarkers for individuals with respiratory tract infections such as TB (81).

These exosomal miRNA sequencing profiles reveal the molecular mechanism of regulating target genes, and of regulation of host physiology and pathology in the process of TB infection by differential miRNAs. These findings provide a comprehensive understanding of miRNAs and repetitive region-derived small RNAs in exosomes during the M. tb infectious process and facilitate the development of potential molecular targets for the detection/diagnosis of TB.

## 5 The roles of other exosomal ncRNAs in *M. tb* infection

CircRNAs are new classes of internal ncRNAs with tissue- and cell-specific expression profiles, and are covalently shut down and extensively expressed in eukaryotes (82). CircRNAs act as miRNA or protein inhibitors (‘sponges’), regulate protein function or self-translate to serve critical biological roles (83, 84). Previous study has revealed that exosomes could carry and protect circRNAs in various body fluids. Exosomal circRNAs in cancers could function at target cells or organs by transporting exosomes, and then participate in the regulation of tumor growth and metastasis (85). Since exosomes are present in diverse body fluids and exosomal circRNAs are highly stable, exosomal circRNAs are potential biomarkers for the diagnosis and prognosis of early and minimally invasive cancer (**Table 2**) (88).

**Table 2:** Summary of exosomal circRNAs from *M. tb* infected subjects (*in vivo* or *in vitro*).

Number	circRNA	Samples	Method screening	Expression pattern	Refs
1	hsa_circ_0129477,hsa_circ_0082641,hsa_circ_0072892, hsa_circ_0104568, hsa_circ_0036372	THP-1 macrophages	RNA sequencing	/	(72)
2	hsa_circRNA_091692,hsa_circRNA_102296, hsa_circRNA_029965, hsa_circRNA_100823	Plasma	RNA sequencing	increase	(86)
3	hsa_circRNA_103571, hsa_circRNA_406755	Plasma	RNA sequencing	decrease	(86)
4	hsa_circ_0000414,hsa_circ_0000681, hsa_circ_0002113, hsa_circ_0002362,hsa_circ_0002908, hsa_circ_0008797, hsa_circ_0063179	PBMCs	RNA sequencing	increase	(87)

/ means “unknown”.

After the exosomal ncRNA profile was analyzed in *M. tb* H37Ra- and *M. bovis* BCG-infected macrophages, circ_0129477, circ_0082641, circ_0072892, circ_0104568 and circ_0036372 were detected and the possible downstream regulatory pathway of these differential circRNAs was revealed (72). The circRNA-miRNA network of interaction implies that a single miRNA can be a target of several circRNAs, while multiple miRNAs can also be targeted by a single circRNA. Yi et al. reported that hsa_circRNA_103571 was markedly decreased in patients with ATB and was involved in the ras pathway, mediation of actin cytoskeleton, and the T- and B-cell receptor pathway, and suggested that circRNA_103571 may be a potential biomarker for ATB identification (86). Qian et al. explored circRNA expression in the peripheral blood of patients with TB using RNA-seq and microarray analysis, and found seven increased circRNAs (circ_0000414, circ_0000681, circ_0002113, circ_0002362, circ_0002908, circ_0008797 and circ_0063179), suggesting that circRNAs may be used as marker molecules to diagnose ATB (87). These studies suggest that the exosomal circRNA signature in TB infection may offer possible targets for the clinical diagnosis of TB. Nevertheless, the roles of circRNAs as biomarkers should be verified in a large sample cluster.

LncRNAs comprise transcripts longer than 200 nucleotides. LncRNAs function as competitive endogenous RNAs by competitively occupying the shared binding sequences of miRNAs, thus sequestering the miRNAs and changing the expression of their downstream target genes (89). Exosomal lncRNAs may also act as biomarkers in the diagnosis of cancers and infectious diseases (90, 91). The present review summarizes all lncRNAs related to TB in following studies (**Table 3**).

**Table 3:** Summary of exosomal lncRNAs from *M. tb* infected subjects (*in vivo* or *in vitro*).

Number	lncRNAs	Samples	Method screening	Expression pattern	Refs
1	LOC152742	Sputum, Plasma	qRT-PCR	increase	(92)
2	NR_038221, NR_003142, and ENST00000570366	Plasma	qRT-PCR	increase	(93)
3	ENST00000422183	Plasma	qRT-PCR	decrease	(94)
4	NR_105053, lncRNAs uc.48+	Plasma	RNA sequencing	increase	(95)
5	ENST00000354432, TCONS_00014296, uc004cov.4, TCONS_00001220, NR_044997, uc002tfi.3, ENST00000442037, ENST00000560602, ENST00000452466, uc001qeg.1, ENST00000483236, ENST00000512284, NR_027391, ENST00000425176, NR_047671, ENST00000420143, NR_029380, ENST00000448001, TCONS_00019631, ENST00000373604, ENST00000584722, ENST00000427151, TCONS_00016358, uc021rro.1, ENST00000546607	Plasma	RNA sequencing	increase	(96)
6	TCONS_00019972, uc001oou.3, ENST00000464125, TCONS_00003870, TCONS_00018420, TCONS_00004316, ENST00000441700, TCONS_00018641, TCONS_00021223, ENST00000439891, NR_051961, NR_027074, ENST00000568137, NR_051961, NR_027074, ENST00000568137, TCONS_00009862, NR_024146, ENST00000584688, NR_024376, ENST00000553496, NR_036546, ENST00000423402, NR_029394, TCONS_00019584, NR_033883, ENST00000397112, ENST00000428188	Plasma	RNA sequencing	decrease	(96)

Evidences demonstrate that lncRNA expression levels are abnormal in PBMCs of patients with TB, implying that lncRNAs are associated with the pathology of TB (97–100). LOC152742 levels in plasma of patients with ATB are higher than those in patients with previous episodes of TB and BCG-vaccinated individuals, suggesting that LOC152742 may be a potential biomarker for ATB discovery and treatment (92). Some small lncRNA sets are highly sensitive and specific in diagnosis. Four DE lncRNAs (NR_038221, NR_003142, ENST-00000570366 and ENST-00000422183) can efficiently separate patients with PTB from controls, with an area under the curve of 0.845 (93). Two lncRNAs (ENST-00000354432 and ENST-00000427151) in the plasma are potential biomarkers for TB diagnosis (94). LncRNAs NR_105053 and uc.48+ were increased in the plasma and may be potential biomarkers to differentiate untreated and cured TB subjects (95). Li et al. found 351 upregulated lncRNAs and 841 downregulated lncRNAs in the serum exosomes of patients with ATB, and NONHSAT-101518.2, NONHSAT-067134.2, NONHSAT-148822.1 and NONHSAT-078957.2 were downregulated in the plasma, suggesting that they may be potential biomarkers for ATB diagnosis (96).

Lv et al. identified mycobacterial transcripts in the exosomes derived from infected macrophages and in serum exosomes of patients with TB. To the best of our knowledge, this was the first report to recognize bacterial RNA in exosomes (101). Gutkin et al. investigated *M. tb* genes in serum exosomes using gene sequencing, and found 2 *M. tb* genes (rrs and rrl) in LTBI and 3 RNA genes (rrs, rrl and Rv2917) in ATB samples (102).

Although exosomal ncRNAs are closely related to TB infection and regulate the corresponding miRNA or mRNA to ultimately mediate transcription or the proteome, their specific regulatory mechanisms and use as diagnostic markers of TB infection need to be further clarified and confirmed in large-scale clinical trials.

## 6 Clinical potential application of exosomal ncRNAs in *M. tb* infection

**Figure 2:**
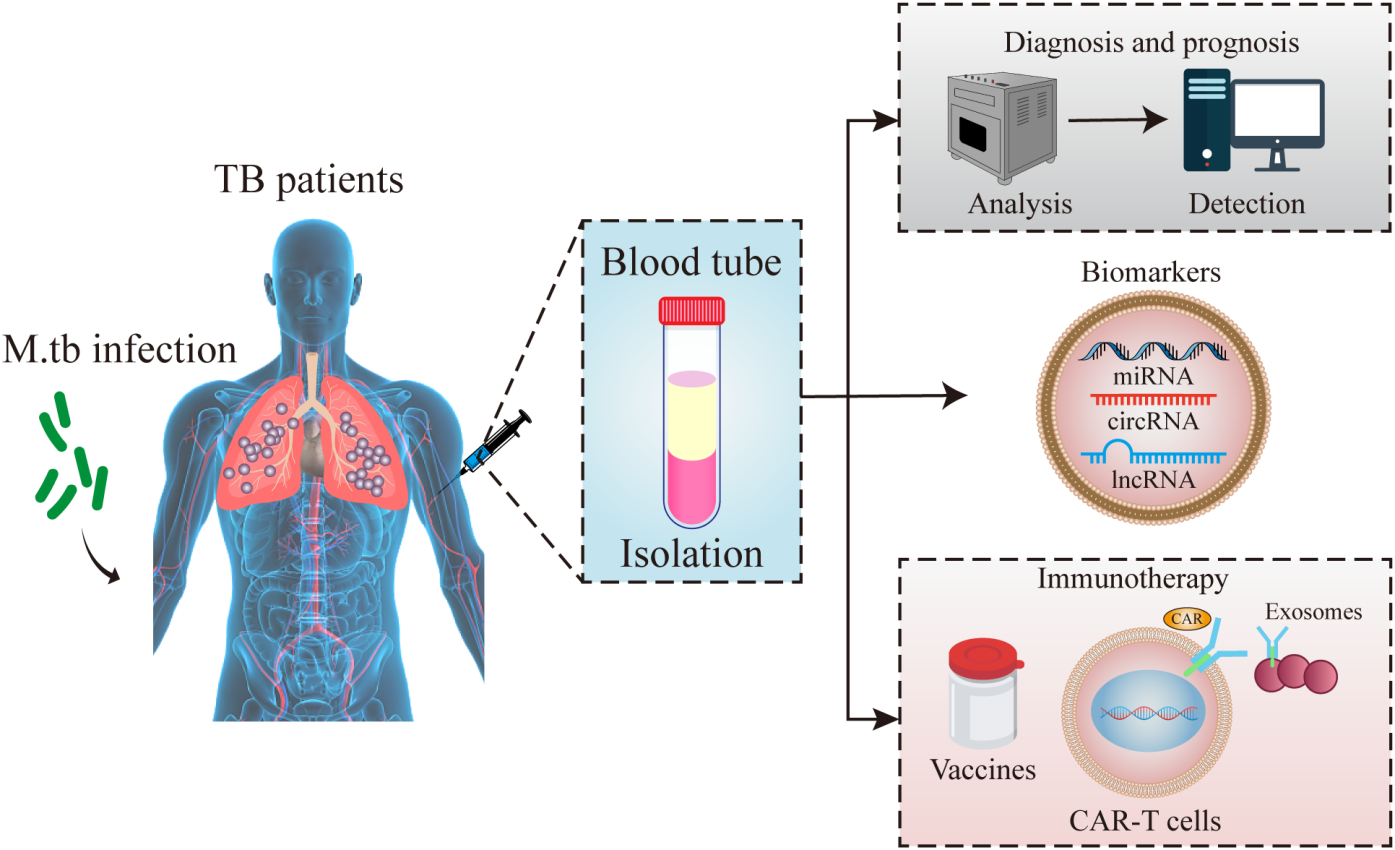
Schematic diagram of exosomes functions. Exosomes in peripheral blood of tuberculosis patients could be used for the diagnostic markers, vaccines and immunotherapy for tuberculosis infection.

Exosomes released from *M. tb* infected macrophages contain pathogen-associated molecular patterns (PAMPs), mycobacteria components lipoarabinomannan and the 19-kDa lipoprotein (103). Moreover, exosomes isolated from *M. bovis* BCG- and *M. tb*-infected macrophages in mice, stimulate the production of TNF-alpha and IL-12 revealing that exosomes promote intercellular communication during an immune response to intracellular pathogens, and exosomes containing PAMPs is an important mechanism of immune surveillance (104). In addition, there was a similar TH1 immune response but a more limited TH2 response in exosome-vaccinated mice compared to BCG-vaccinated mice suggesting that exosomes might serve as a novel cell-free vaccine against an *M. tb* infection (30). These documents show that exosomes cloud be used as an immunotherapy or vaccine to prevent tuberculosis infection in clinic (**Figure 2**). This study is mainly aimed at exosomes as a biomarker for the diagnosis of tuberculosis infection, especially for ncRNAs in exosomes.

Screening approaches based on biomarkers (specially quantified in blood specimens) help detect subjects with a higher risk of developing ATB, or at early stages of disease (105, 106). To satisfy these requirements, some clinical studies have recognized molecular signatures using PCR in blood samples, which were demonstrated to be effective in prospective cohorts. Such studies utilize gene or protein expression screening approaches (microarrays, RT-qPCR and RNA-seq) to find consistent changes related to specific clinical features, typically comparing patients with TB with patients with LTBI or healthy individuals, and verifying a short-list of chosen candidate markers in follow-up research of TB groups to forecast who will suffer pulmonary disease (107–109). Numerous signatures have been suggested that were more sensitive and specific than the existing screening assays (110–113). In a similar manner, signatures of genes or proteins that are expressed differently in response to TB therapy can assist in clinical follow-up assessment or support prognostic assays of new TB cases (114).

As for cancers and infectious diseases, miRNAs are considered as a probable source of such biomarkers, bringing about numerous discovery reports, which either screen subject-vs-control cohorts or aim to confirm exosomal ncRNAs found in experiments (115). As aforementioned, *M. tb* infection induces the abnormal expression of genes (including ncRNAs) involved in immune, inflammation, autophagy and apoptosis pathways that can be potential diagnostic or prognostic biomarkers of disease and therapy response. Pedersen et al. have attempted to find known ncRNA signatures that can be assayed in accessible specimens, focusing on circulating ncRNAs, which were measured in blood (PBMCs or serum/plasma) (116). The expected ncRNA signature should specifically find *M. tb*-infected subjects, and may separate an active infection from LTBI.

The amount of exosomal biomolecules in the blood is difficult to detect. Compared with proteins and lipids, exosomal ncRNAs can be amplified by RT-qPCR, and thus, is more frequently used in the clinic and assayed (117). Due to inclusion in exosomes, ncRNA degradation can be prevented and ncRNAs may be a source of stable RNAs that can be used as disease biomarkers (118). Their enrichment and high stability in exosomes may permit their noninvasive detection in body fluids (119, 120). Therefore, ncRNAs in exosomes may be used in diagnosis, as biomarkers and for therapy in TB infection (**Figure 2**).

## 7 Conclusions

In *M. tb* infection, exosomes from the infected immune cells exert inherent immune regulation effects on anti-TB immunity. Therefore, exploring potential specific components and biological functions of exosomes is helpful in developing novel diagnosis and therapy strategies. Different ncRNA molecules have been found in exosomes after *M. tb* infection, which shed new light on the potential role of exosomal ncRNAs as novel TB biomarkers for developing the next generation of TB diagnostic strategies. Exosomes have shown strong potential in delivering vaccine components (proteins, peptides and RNA) in different infectious diseases, showing the potential to provide a more effective vaccine strategy for TB.

## Author contributions

YL, JWu, and NW conceived the work and wrote the manuscript. JWa and LT designed and revised the manuscript. XY and YJ discussed and edited the manuscript. All authors contributed to the article and approved the submitted version.
